# Human Microbiome in Children, at the Crossroad of Social Determinants of Health and Personalized Medicine

**DOI:** 10.3390/children8121191

**Published:** 2021-12-16

**Authors:** Talía Sainz, Valeria Pignataro, Donato Bonifazi, Simona Ravera, María José Mellado, Antonio Pérez-Martínez, Adela Escudero, Adriana Ceci, Cristina Calvo

**Affiliations:** 1 Hospital La Paz, Pº Castellana 261, 28046 Madrid, Spain; mariajose.mellado@salud.madrid.org (M.J.M.); aperezmartinez@salud.madrid.org (A.P.-M.); adela.escudero@salud.madrid.org (A.E.); ccalvorey@gmail.com (C.C.); 2 La Paz Hospital Reserach Institute (IdiPAZ), Pº Castellana 261, 28046 Madrid, Spain; 3 CIBER de Enfermedades Infecciosas (CIBERInfec), Instituto de Salud Carlos III (ISCIII), 28029 Madrid, Spain; 4 Consorzio per Valutazioni Biologiche e Farmacologiche, Via N. Putignani n. 178, 70122 Bari, Italy; vpignataro92@gmail.com (V.P.); dbonifazi@cvbf.net (D.B.); AdriCeci.uni@Gmail.com (A.C.); 5 TEDDY European Network of Excellence for Paediatric Research, Via Luigi Porta 14, 27100 Pavia, Italy; 6 PHArmaceutical Research Management SRL, Via Albert Einstein, 26900 Lodi, Italy; sravera@cvbf.net; 7 Departamento de Pediatría, Universidad Autónoma de Madrid (UAM), 28049 Madrid, Spain

**Keywords:** child, social determinants of health, human microbiome, personalized medicine

## Abstract

The evolving field of microbiome research offers an excellent opportunity for biomarker identification, understanding drug metabolization disparities, and improving personalized medicine. However, the complexities of host–microbe ecological interactions hinder clinical transferability. Among other factors, the microbiome is deeply influenced by age and social determinants of health, including environmental factors such as diet and lifestyle conditions. In this article, the bidirectionality of social and host–microorganism interactions in health will be discussed. While the field of microbiome-related personalized medicine evolves, it is clear that social determinants of health should be mitigated. Furthermore, microbiome research exemplifies the need for specific pediatric investigation plans to improve children’s health.

## 1. Introduction

The evolving field of the microbiome has revolutionized biomedical research in recent years, where it has emerged as an independent research specialty. In medicine, applied microbiome research offers new opportunities, including biomarker discovery, development of therapeutic targets, understanding of disparities in drug metabolization, and avenues for personalized medicine [1]. However, the complexities of the host–microbe ecological interactions hinder clinical transferability.

Personalized medicine (PM), also called precision or individualized medicine, is a promising field that may help clinicians determine which medical treatments will work best for each patient [2]. The simplest definition would be “the provision of the right treatment to the right patient at the right dose at the right time” [3]. As not all patients respond in the same way to a given therapy, PM has the potential to make medical practice more efficient based on genetic, biological, or psychosocial characteristics [4,5,6]. All these factors are to be taken into account for medical management, together with patient preferences.

Research in pharmacogenomics, proteomics, and metabolomics has already led to the identification of several genes, mRNA, proteins, and metabolites that can act as biomarkers and reliably reflect inter-individual variability in disease expression, with the potential to predict outcomes. Different biomarkers are being tested in experimental studies, but only a few have been already integrated into clinical practice [7]. Today, the microbiome is envisioned as one of the most critical and hypothetically modifiable markers of disease [1].

While the evidence supporting the interaction between the human host and the microbiome increases, the identification of microbiome-related biomarkers and the understanding of the role of microbiota in drug metabolization are also the focus of intense research. However, whether genetic or related to microbiota, validation of biomarkers among adults does not necessarily imply its usefulness in children, as gene expression and microbial colonization vary during childhood. It is known that the microbiome is established mainly during the first year of life, although fluctuations in the ecosystem occur over time together with lifetime changes [8]. Beyond age and genetic factors, the microbiome is deeply influenced by geographical, dietary, and lifestyle-related factors [9]. Studies suggest that these factors may be especially relevant in shaping the microbiome during childhood [7,8]. Social determinants of health have a direct impact on undoubtfully critical factors for health, such as malnutrition, access to treated water, or health care. In comparison, their effect on microbiota composition and how much these changes may contribute to health and disease may seem trivial and has not been well established.

## 2. Social Determinants of Health and the Microbiome in Children

Social inequities, poverty, or racism have profound impacts on life expectancy [3]. Defined by the World Health Organization as the “conditions in which people are born, grow, work, live, and age and the wide set of forces and systems shaping the conditions of daily life” [10], the so-called social determinants of health (SDOH) are known to have a powerful effect on health outcome [11,12,13]. Disparities among children’s health and healthcare utilization along demographic lines such as race and income have long been documented as factors influencing children’s morbidity and mortality [10]. Although SDOH influence health and well-being across individuals of all ages, in children and young people, physical, social, and emotional capabilities that develop early in life provide the basis for life course health and well-being [14]. Emerging data demonstrate that exposure to violence, food scarcity, poverty, and lack of housing, as well as race, ethnicity, gender, education, and health literacy, are potent determinants and comorbid issues for many conditions [13]. Recently, the human microbiome has been identified as a potentially modifiable determinant of health, which is highly determined itself by social and geographical conditions.

Although our understanding of the impact of the human microbiome on health is still in the early stages, current knowledge indicates that the interaction between microbiota and the host is strong. Around 10^13^ microorganisms, including bacteria, viruses, fungi, and protozoa, accounting for a total mass of 0.2 kg [15], inhabit our bodies and constitute the human microbiome. An overwhelming amount of data has underlined the influence of the first years of life to shape the microbiome–immune system interactions in recent years. Very early in life, the microbiome is first established by the colonization of microorganisms from the mother’s skin, genital tract microbiota, breast milk, and after the introduction of complementary feeding [16,17]. Factors related to the mode of delivery, antimicrobial exposure early in life, or breast-feeding duration have been shown to impact microbiota acquisition [8,16]. Furthermore, it is known that these factors condition variations in gut microbiota that are associated with an increased risk of suffering from allergic diseases, asthma, celiac disease, or inflammatory bowel disease [17,18]. Geographic location and ethnicity also determine variability in the ecosystem [19], together with well-known dietary and lifestyle-related factors [17].

These environmental factors are deeply related to socio-economic conditions, including dietary restrictions, hygiene habits, housing conditions, and access to treated water or health care. Dietary habit modifications in the course of migration, for example, are key in shaping the gut microbiota. An ecosystem enriched in microorganisms specialized in degrading fibers, characteristic of children living in limited-resource settings, can be an adaptation to enhance energy obtention from the diet but could turn deleterious in the presence of a westernized diet [20].

The geographical variability of vaccine response is another compelling example of the critical implications of host–microbiome and environmental interactions [21]. In low-income countries, the poorer immunization rates achieved by oral vaccines (cholera, poliovirus, and rotavirus) have been classically related to environmental, socio-economic, or nutritional conditions [22]. However, it may also be explained by changes in the intestinal microbiota composition [21]. In a kind of infinite loop, dietary factors have a clear impact on the microbiome. Still, changes in the microbiome can lead to behavioral adaptations, leading to a subsequent modification in dietary habits [23]. Among other challenges in the field, causality is always questionable in most microbiome studies in humans. Autism spectrum disorder (ASD), common comorbidities of which are functional gastrointestinal disorders [24], is an excellent example of this bidirectionality. Manipulation of the gut microbiome could offer a promising treatment option for children with ASD. Recently, significant changes in serum neurotransmitters and an improvement in behavioral and gastrointestinal symptoms were observed during a fecal microbiota transplantation trial in children diagnosed with autism [25]. Yet, the longitudinal follow-up would be essential for further understanding of the so-called “gut-brain” axis.

The fact that the gut microbiome in children living in resource-limited settings has remained underreported in microbiome research is another clear example of how social inequities impact health from the very primary step of knowledge generation. Nevertheless, consistent data illustrate how pathogenic species are often detected in higher abundance among malnourished children living in low-income settings [19,26]. While there is agreement that nutrition and gut microbiota are linked, particularly in vulnerable populations such as children, it is highly controversial to what extent the theoretically modifiable human microbiome is a potential therapeutic target. Fecal microbiota transplantation has shown efficacy in very limited settings (recurrent *Clostridioides difficile* associated diarrhea). Still, studies addressing the role of microbiota modulation with probiotics, prebiotics, or dietary interventions in treating and recovering from infections or inflammatory diseases have raised controversial results [27,28,29]. Although these treatments’ impact and therapeutic potential have not been well-established yet, the evidence supports the need to implement measures to prioritize food security worldwide. Making nutritional modifications in areas with limited resources is a challenge, but it is also a priority to improve health.

**Figure 1:**
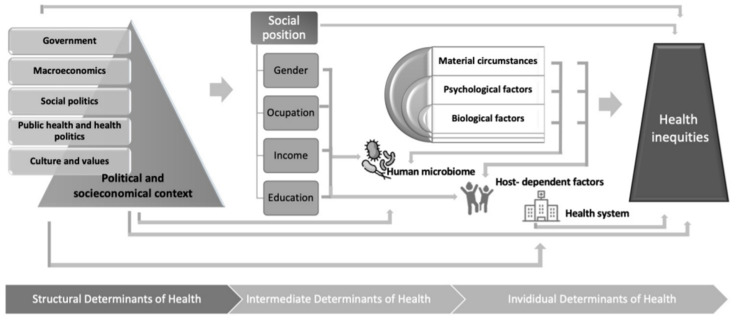
Human microbiome at the crossroad between social determinants of health and personalized medicine.

Untangling the crossroad of SDOH, the human microbiome, and human health is a formidable challenge (Figure 1). Unfortunately, because the individual’s microbiota is fundamentally established during the first three years of life (from childbirth to the consolidation of the adult diet) [17], the impact of socio-economic factors on the microbiome composition might be more significant in children compared to adults. An “unfavorable” microbiota may cause lasting damage [20,30]. Hence, the idea of the bidirectionality of social and host–microorganism interactions in health should be integrated into research and clinical perspectives from today. In addition to the possible therapeutic implications, some of them already mentioned, modification of the microbiota in childhood has been postulated to be key in the prevention of infections [31], allergy [32], asthma [33], or even cancer in childhood [34,35,36]. Therefore, the inclusion of children in clinical trials evaluating dietary modifications and their impact on various diseases and overall health should be prioritized.

## 3. Personalized Medicine and the Microbiome

Therapeutic efforts to target the microbiome have shown, as mentioned, contradictory results [37,38,39]. Modulation of the microbiota with probiotics, prebiotics, dietary interventions, or even fecal microbiota transplantation is under research. However, apart from the treatment of *Clostridioides difficile*-associated diarrhea, there have been few impacts in terms of clinical practice outside research. Beyond the hospital environment, nevertheless, diet is increasingly appreciated to have a tremendous impact on many aspects of life, including health and disease. Personalized nutrition aims to characterize inter-individual host and microbiome variations and generate data-driven personalized dietary recommendations [40] and is becoming more and more popular worldwide. The concept would be as follows: first, the diet gives rise to a specific microbiota for each person. Second, characterization of the individual’s microbiome and gut-derived metabolites would be directed towards a personalized nutrition plan.

This holistic approach does not seem feasible for disease treatment, where personalized medicine requires the identification of key proteomic, metabolomic, or microbiome-related biomarkers. In fact, the search for biomarkers that allow anticipating and monitoring disease has been a constant in medical research. The potential applications of microbiota-related biomarkers and metabolic/immune check-points in all areas of medicine, from obesity to cancer, are endless. Most attempts to personalize the approach based on microbiome contributions to human health come from research in diabetes [41], cardiovascular disease [42], metabolic syndrome/obesity [43], and cancer [44]. Short-chain fatty acids (SCFAs) such as butyrate, acetate, or propionate [45] are critical drivers of T-cell subset proliferation and activity [46], and they are produced after fermentation of complex dietary carbohydrates by gastrointestinal bacteria. These metabolites have been suggested to influence both maternal and newborn down-regulation of pro-inflammatory responses, playing an essential role in tolerance phenomena and atopy and asthma in childhood [47].

The diagnostic and therapeutic potential of SCAs is being explored in different settings, from allergies to autoimmune disorders such as systemic lupus erythematosus [48]. In addition, SCFAs and other microbiota-derived metabolites such as lipopolysaccharides, beta-cresol, and bacterial toxins in blood and urine are being explored as diagnostic tools and for early intervention in autism spectrum disorder [49]. In this context, despite the uncertainty regarding the cause-to-effect relationship, the diagnostic potential is appealing, and the design of personalized treatments seems promising. The tumor microenvironment is another focus of intense research nowadays. In colorectal cancer, gut microbiota biomarkers have gained attention for their potential for early non-invasive diagnosis, with good sensitivity, specificity, and even cost-effectiveness [50]. A similar approach has been made in esophageal cancer [51], and clinical trials are ongoing for fecal microbiota transplantation as an immunomodulatory strategy to improve response to treatment among advanced lung cancer patients [52].

The future clinical relevance of the microbiome and microbiota-derived compounds as new biomarkers for diagnosis and targeted interventions in medicine is unclear. Still, it represents an important proportion of options being explored today for personalized medicine. Unfortunately, most of these investigations will not be transferable to children, as the evolving nature of the microbiome impairs us from extrapolating adults’ data. To bring the promise of personalized medicine to children, specific pediatric investigation plans that contemplate the design of studies aimed at typical childhood pathologies and the inclusion of children in clinical trials are required. Plasticity might be more remarkable in children in terms of targeting the microbiome, and achieving long-lasting effects might be easier in a non-established ecosystem, making it even more important to prioritize pediatric investigation in the field. Longitudinal studies, together with new tools for a functional approach to the microbiome and an ecological perspective, are required to deepen our understanding of such a complex field.

## 4. Conclusions

To what extent the study of the microbiome will have clinical implications is still an unanswered question, but promising research points towards the development of a microbiome-based personalized medicine. Microbiome composition is deeply influenced by socio-economic factors, especially during childhood. While the field of microbiome-related personalized medicine evolves, it is clear that SDOH can and should be mitigated. Improving living conditions and fighting the unequal distribution of power, money, and resources is crucial for children. Due to the evolving nature of the microbiome, children will need to be the focus of research if the aim is to bring personalized medicine to pediatrics.

## Data Availability

Not applicable.
